# Enrollment Patterns of Medicare Advantage Beneficiaries by Dental, Vision, and Hearing Benefits

**DOI:** 10.1001/jamahealthforum.2023.4936

**Published:** 2024-01-12

**Authors:** Avni Gupta, Diana Silver, David J. Meyers, Genevra Murray, Sherry Glied, José A. Pagán

**Affiliations:** 1 Department of Public Health Policy and Management, School of Global Public Health, New York University, New York, New York; 2 Department of Health Services, Policy, and Practice, Brown University School of Public Health, Providence, Rhode Island; 3 Robert F. Wagner Graduate School of Public Service, New York University, New York, New York; 4 Brookings Institution, Washington, DC

## Abstract

**Importance:**

Most Medicare beneficiaries now choose to enroll in Medicare Advantage (MA) plans. Racial and ethnic minority group and low-income beneficiaries are increasingly enrolling in MA plans.

**Objective:**

To examine whether dental, vision, and hearing supplemental benefits offered in MA plans are associated with the plan choices of traditionally underserved Medicare beneficiaries.

**Design, Setting, and Participants:**

This exploratory observational cross-sectional study used data from the 2018 to 2020 Medicare Current Beneficiary Survey linked to MA plan benefits. The nationally representative sample comprised primarily community-dwelling MA beneficiaries enrolled in general enrollment MA plans. Data analysis was performed between April and October 2023.

**Exposures:**

Beneficiary self-identified race and ethnicity and combined individual and spouse income and educational attainment.

**Main Outcomes and Measures:**

Binary indicators were developed to determine whether beneficiaries were enrolled in a plan offering any dental, comprehensive dental, any vision, eyewear, any hearing, or hearing aid benefit. Mixed-effects logistic regression models were estimated to report average marginal effects adjusted for beneficiary-level demographic and health characteristics, plan attributes, and plan availability.

**Results:**

This study included 8139 (weighted N = 31 million) eligible MA beneficiaries, with a mean (SD) age of 77.7 (7.5) years. More than half of beneficiaries (54.9%) were women; 9.8% self-identified as Black, 2.0% as Hispanic, 83.9% as White, and 4.2% as other or multiple races or ethnicities. Plan choices by dental benefits were examined among 7516 beneficiaries who were not enrolled in any dental standalone plan, by vision benefits for 8026 beneficiaries not enrolled in any vision standalone plan, and by hearing benefits for 8131 beneficiaries not enrolled in any hearing standalone plan. Black beneficiaries were more likely to enroll in plans with any dental benefit (9.0 percentage points [95% CI, 3.4-14.4]; *P* < .001), any comprehensive dental benefit (11.2 percentage points [95% CI, 5.7-16.7]; *P* < .001), any eye benefit (3.0 percentage points [95% CI, 1.0 to 5.0]; *P* = .004), or any eyewear benefit (6.0 percentage points [95% CI, 0.6-11.5]; *P* = .03) compared with White beneficiaries. Lower-income individuals (earning ≤200% of the federal poverty level) were more likely to enroll in a plan with a comprehensive dental benefit (4.4 percentage-point difference [95% CI, 0.1-7.9]; *P* = .01) compared with higher-income beneficiaries. Beneficiaries without a college degree were more likely to enroll in a plan with a comprehensive dental benefit (4.7 percentage-point difference [95% CI, 1.4-8.0]; *P* = .005) compared with those with higher educational attainment.

**Conclusions and Relevance:**

The results of this study suggest that racial and ethnic minority individuals and those with lower income or educational attainment are more likely to choose MA plans with dental or vision benefits. As the federal government prepares to adjust MA plan star ratings for health equity, implements MA payment cuts, and allows increasing flexibility in supplemental benefit offerings, these findings may inform benefit monitoring for MA.

## Key Points

**Question:**

Do racial and ethnic minority group and lower-income Medicare Advantage (MA) beneficiaries prefer plans with dental, vision, or hearing benefits?

**Findings:**

In this cross-sectional study of 8139 MA beneficiaries (weighted N = 31 million), non-Hispanic Black individuals, individuals with lower income, and individuals with lower educational attainment were more likely to enroll in plans offering dental or vision benefits.

**Meaning:**

Currently, racial and ethnic minority individuals constitute a disproportionate share of MA enrollees; forthcoming policies, such as payment cuts and Health Equity Index use, may lead plans to change their included supplemental benefits, which may alter enrollment patterns across racial and ethnic groups.

## Teaser

This cross-sectional study examines the association of dental, vision, and hearing supplemental benefits offered in Medicare Advantage plans with plan choices of traditionally underserved Medicare beneficiaries.

## Introduction

Medicare Advantage (MA) now enrolls more than half of all Medicare beneficiaries^1^ and is experiencing increased enrollment of racial and ethnic minority group and dual-eligible beneficiaries.^2^ For example, between 2009 and 2018, MA enrollment increased 66% among Black beneficiaries and 46% among White beneficiaries.^2^ Among other value propositions of MA, such as an annual out-of-pocket maximum, MA plans can offer supplemental benefits beyond traditional Medicare (TM) Part A and B benefits.^3^ Supplemental benefits are funded by rebate dollars paid by the US Centers for Medicare & Medicaid Services (CMS) to MA plans according to the bid-benchmark and quality bonus program. In 2022, rebates reached nearly $20 billion.^4^ Plans are required to use these rebate dollars for supplemental benefits or for decreased cost sharing or Part B premiums.^5^ Dental, vision, and hearing are the 3 most conventional supplemental benefits and are now offered by more than 90% of MA plans.^6^ Dental, vision, and hearing benefits have attracted substantial attention from policy makers, owing to both political pressure^7^ and evidence of high need within the Medicare population. The cost of adding these benefits to TM is estimated to be $358 billion.^8^ Almost all adults aged 65 years or older (96%) have had a cavity, 1 in 5 have untreated tooth decay, 2 in 3 have a gum disease, 1 in 5 are completely edentulous and often need dental examinations or more comprehensive care for dentures or implants,^9^ 44% report hearing difficulties,^10^ and 35% report vision difficulties.^10^ In addition to increasing enrollment of racial and ethnic minority beneficiaries and low-income beneficiaries in MA plans,^11^ previous studies also reported that a higher percentage of racial and ethnic minority beneficiaries and low-income beneficiaries are enrolled in Special Needs Plans (SNPs), zero-premium plans, and low-star plans.^2^ However, to our knowledge, no prior studies have examined enrollment patterns of beneficiaries by dental, vision, and hearing benefits.

Our study examines the role of dental, vision, and hearing benefits in enrollment patterns of traditionally underserved MA beneficiaries, such as racial and ethnic minority individuals, lower-income individuals, and individuals with lower educational attainment, who often face economic, cultural, or linguistic barriers to accessing health services or lack familiarity with the health care system.^12^ Moreover, unlike the standard Part A and B benefits, each plan can decide the generosity of their supplemental benefit offerings, creating a wide heterogeneity in the generosity of MA plan supplemental benefits. We examined sorting patterns by any benefit or by comprehensive benefits. Knowledge of sorting patterns of traditionally underserved MA beneficiaries by the most common supplemental benefits in MA may help inform decisions about benefit regulations by policymakers^13,14^ in the context of recent and forthcoming policy changes in the MA program, such as plan payment cuts,^15^ linking of plan payments to equity through the Health Equity Index,^16^ and increasing flexibility in supplemental benefit offerings.^6,17^

## Methods

### Data Source

We conducted this exploratory observational cross-sectional study using a national sample of MA and TM beneficiaries in the Medicare Current Beneficiary Survey (MCBS) from 2018 to 2020. The MCBS is an annual nationally representative survey of MA and TM beneficiaries with a rotating cohort design that samples nearly 15 000 beneficiaries each year. The most recent response rate was 67.0% for the baseline interview and 86.4% for the following interviews.^18^ We linked beneficiary-level MCBS data with publicly available MA plan benefit, landscape, and penetration data using MCBS respondent county of residence and MA contract and plan numbers. This study was deemed exempt from human participant review by the New York University Institutional Review Board. We obtained the MCBS data set from the CMS according to a data use agreement, which prohibits identification of individual beneficiaries; hence, informed consent procedures were not applicable. The study followed the Strengthening the Reporting of Observational Studies in Epidemiology (STROBE) reporting guideline.

### Sample

We analyzed data for MCBS respondents enrolled in the same general enrollment MA plan^19^ with Part A and B benefits for all 12 months in the survey year. We excluded enrollees in dual-eligible SNPs or integrated Medicare-Medicaid plans because their dental, vision, and hearing benefit designs are determined by state Medicaid policies.^19,20^ We excluded beneficiaries with high and unique health care needs, those aged younger than 65 years,^21,22^ individuals with Medicare eligibility due to a disability,^21^ and enrollees of chronic condition and institutional SNPs.^21,22^ After these exclusions, the sample included 195 facility-dwelling and 7944 community-dwelling beneficiaries. We further excluded beneficiaries enrolled in non-MA standalone plans for the respective benefit and assumed that all beneficiaries surveyed in 2018 were not enrolled in a hearing standalone plan, as MCBS did not report these data in 2018 (eFigure 1 in Supplement 1).

### Dependent Variables

Beneficiaries were classified as being enrolled in a plan with at least 1 dental benefit (or ≥1 preventive dental benefit because all beneficiaries enrolled in a plan with any dental benefit had a preventive dental benefit, including oral examinations, prophylaxis, dental radiographs, and fluoride treatment), at least 1 comprehensive dental benefit (nonroutine services, diagnostics, restorative, endodontics, periodontics, prosthodontics, tooth extractions, or oral and maxillofacial surgery), and the number of dental benefits in the plan. For vision, the benefit categories were at least 1 eye benefit (or ≥1 eye examination benefit because 99.9% of beneficiaries with any eye benefit also had an eye examination benefit), at least 1 eyewear benefit, and the number of eye benefits. For hearing, the benefit categories were at least 1 hearing benefit (or ≥1 hearing examination benefit because 98.7% of beneficiaries with any hearing benefit also had a hearing examination benefit), at least 1 hearing aid benefit, and the number of hearing benefits. We only included mandatory benefits. The MA plans can choose to provide a supplementary benefit to all plan enrollees (mandatory benefits) or to only those plan enrollees who pay an additional premium (optional benefits). It was not possible to determine through our data which beneficiaries paid a premium for optional benefits.

### Independent Variables

To examine the enrollment patterns of traditionally underserved MA beneficiaries, we categorized beneficiaries by race and ethnicity, educational attainment (no college degree or college degree or higher),^21^ and combined individual and spouse income according to federal poverty level (FPL; ≤200% or >200%). Participants self-identified as Hispanic, non-Hispanic Black, non-Hispanic White, or other or multiple races or ethnicities (including American Indian or Alaska Native or Asian).

### Covariates

We determined the following characteristics of respondents: age (65-74, 75-84, or ≥85 years), marital status (married or not married, including beneficiaries reporting being widowed, divorced, separated, living alone, or never married), sex (male or female), rural-urban residence based on Rural-Urban Commuting Area Codes for Census tract, self-reported health status (good to excellent or fair to poor), chronic illness burden (count of ever being diagnosed with myocardial infarction, congestive heart failure, stroke, coronary heart disease, nonskin cancer, depression, emphysema, asthma, chronic obstructive pulmonary disease, or diabetes; categorized as 1, 2, or >2 vs 0 chronic conditions),^23,24^ and functional limitations (counts of limitations in any instrumental activities of daily living [IADLs] or activities of daily living [ADLs]; categorized as only IADLs, 1-2 or 3-6 ADLs, or 0 limitations).^23,24^ We controlled for plan characteristics potentially relevant to enrollment decisions^22,23,25,26,27^ and focused on those available on the Medicare Plan Finder website, including categorical variables for coverage type (health maintenance organization, preferred provider organization, or private-fee-for-service), monthly Part C and D premiums minus rebates ($0, ≤$51, or >$51), maximum in-network Part C out-of-pocket costs (<$3500, $3501-$4950, $4951-$6700, or >$6700 based on 25th, 50th, and 75th percentiles), and overall Part C and D star rating (<4 or ≥4 stars). All beneficiaries in our sample had Part D benefits.

### Statistical Analysis

We used mixed-effects multivariable logistic regression models with county-level random intercepts to estimate within-county adjusted odds of enrollment in a plan with a certain benefit. By adjusting for county, our study eliminates confounding due to differences in plan availability across counties.^25^ We compared beneficiary characteristics by plan benefits using the adjusted Wald test. Results from multivariable models are reported as marginal effects, which can be interpreted as percentage-point differences in the conditional likelihood of the dependent variable associated with a 1-unit change in the independent variables. We used mixed-effects negative binomial regression to estimate the incidence rate ratio (IRR) of the number of benefits across independent variables.

We considered alternative model specifications to account for county-level MA plan offerings. We included county indicators (fixed effect), restricting our sample to counties with at least 50 observations (59 of 119 counties were eligible). We also included indicators for tertiles of county-level MA penetration, and we conducted additional sensitivity analyses including beneficiaries with standalone plans and with high needs (aged <65 years or enrolled in a chronic condition or institutional SNP). Finally, we used conditional logistic regression to account for plan-choice differences by county.

All analyses were conducted in Stata SE, version 17.0 (StataCorp LLC), and incorporated weights to account for the complex MCBS survey design and intrapersonal correlation. Because this study aimed to identify determinants of plan selection, statistical significance was set at *P* < .05 and hypothesis tests were 2 sided. Data analysis was performed between April and October 2023.

## Results

### Sample Characteristics

Our overall national sample included 8139 MA beneficiaries (weighted N = 31 million), with a mean (SD) age of 77.7 (7.5) years. More than half of beneficiaries (54.9%) were women compared with men (45.0%). Beneficiaries self-identified as Black (9.8%), Hispanic (2.0%), White (83.9%), or other or multiple races or ethnicities (4.2%). A total of 8.5% of beneficiaries were enrolled in a dental standalone plan, 1.6% in a vision standalone plan, and 0.1% in a hearing standalone plan (eTable 1 in Supplement 1).

Our analytic sample was limited to individuals without the respective standalone plan: that is, to 7516 beneficiaries for an analysis of enrollment patterns by dental benefits, to 8026 beneficiaries for vision benefits, and to 8131 beneficiaries for hearing benefits (eTable 2 in Supplement 1). The overall sample and the sample for each benefit were similar in their beneficiary mix. More than 83.4% of beneficiaries were White, 40.0% had higher income (≥200% of the FPL), and 65.0% did not complete a college degree (eTable 2 in Supplement 1). We observed that a higher percentage of racial and ethnic minority beneficiaries, individuals with lower income, and beneficiaries with less educational attainment were enrolled in plans with a dental, vision, or hearing benefit (Table and eTable 3 in Supplement 1). For example, 77.1% of Black beneficiaries and 68.3% of White beneficiaries were enrolled in a plan with a dental benefit. A total of 96.7% of Black beneficiaries and 95.5% of White beneficiaries were enrolled in a plan with an eye benefit. Finally, 88.2% of Black beneficiaries and 85.4% of White beneficiaries were enrolled in a plan with a hearing benefit (Table).

**Table.** Characteristics of Medicare Advantage Beneficiaries by Enrollment in a Plan With or Without a Dental, Vision, or Hearing Benefit, 2018 to 2020^a^

Characteristic	Weighted % enrolled in a Medicare Advantage plan (95% CI)					
	Dental (n = 7516)		Vision (n = 8026)		Hearing (n = 8131)	
	With benefit	Without benefit	With benefit	Without benefit	With benefit	Without benefit
Overall	68.5 (63.9-72.8)	31.5 (27.2-36.1)	95.8 (94.2-96.9)	4.2 (3.0-5.8)	85.4 (81.7-88.4)	14.6 (11.5-18.3)
Race and ethnicity						
Hispanic	67.7 (57.8-76.2)	32.3 (23.8-42.2)	99.3 (95.2-99.9)	0.7 (0.1-4.8)	88.0 (80.5-92.9)	12.0 (7.1-19.5)
Non-Hispanic Black	77.1 (70.6-82.6)	22.8 (17.3-29.4)	96.7 (92.0-98.7)	3.3 (1.3-8.0)	88.2 (83.6-91.6)	11.8 (8.4-16.4)
Non-Hispanic White	68.3 (63.7-72.6)	31.7 (27.4-36.3)	95.5 (93.6-96.9)	4.5 (3.1-6.4)	85.4 (81.4-88.7)	14.6 (11.3-18.6)
Other or multiple^b^	52.9 (40.9-64.4)	47.1 (35.5-59.0)	97.3 (91.5-99.2)	2.6 (0.8-8.5)	77.6 (66.4-85.8)	22.4 (14.2-33.6)
Income level, % FPL						
≤200	69.4 (64.7-73.7)	30.5 (26.2-35.2)	95.0 (93.0-96.4)	5.0 (3.5-7.0)	85.1 (81.3-88.2)	14.9 (11.8-18.7)
>200	67.8 (62.7-72.5)	32.2 (27.5-37.3)	96.3 (94.8-97.4)	3.7 (2.6-5.2)	85.6 (81.5-88.9)	14.4 (11.1-18.4)
Educational attainment^c^						
No college degree	70.4 (66.1-74.4)	29.5 (25.5-33.9)	95.3 (93.5-96.6)	4.7 (3.4-6.5)	85.9 (82.0-89.1)	14.1 (10.9-18.1)
College degree or higher	64.2 (57.7-70.2)	35.8 (29.8-42.2)	96.9 (95.3-97.9)	3.1 (2.1-4.7)	84.5 (80.5-87.8)	15.5 (12.2-19.5)

Abbreviation: FPL, federal poverty level.

^a^ Weighted percentages represent the row percentage.

^b^ This group comprised individuals who identified as American Indian or Alaska Native or Asian or as multiple races or ethnicities.

^c^ Educational attainment was missing for 1.9% of beneficiaries; row percentages may not add to 100% because of rounding.

### Multivariable Models

#### Dental Benefits

**Figure 1.**
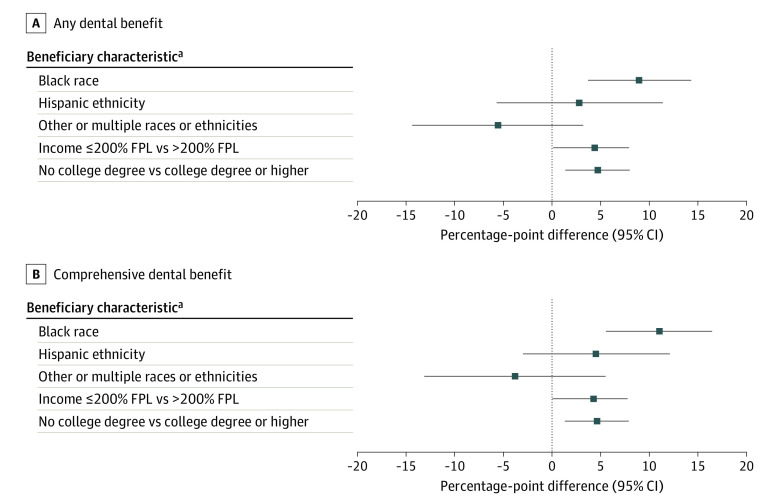
Average Adjusted Percentage-Point Difference in Enrollment Among Beneficiaries by Dental Benefits Adjusted marginal effects can be interpreted as adjusted percentage-point difference in enrollment and were derived from mixed-effects multivariable logistic regression models that included county-level random effect, beneficiary race and ethnicity, socioeconomic and demographic characteristics, rural-urban residence, health status, chronic illness burden, functional limitations, and plan features. FPL indicates federal poverty level. ^a^White race, higher income, and higher educational attainment served as the comparators.

Black beneficiaries were more likely to enroll in a plan with any dental benefit (9.0 percentage points [95% CI, 3.4-14.4]; *P* < .001) or with any dental comprehensive benefit (11.2 percentage points [95% CI, 5.7-16.7]; *P* < .001) compared with White beneficiaries. Lower-income individuals (earning ≤200% of the FPL) were more likely to enroll in a plan with a comprehensive dental benefit (4.4 percentage-point difference [95% CI, 0.1-7.9]; *P* = .01) compared with higher-income beneficiaries. Beneficiaries without a college degree were more likely to enroll in a plan with a comprehensive dental benefit (4.7 percentage-point difference [95% CI, 1.4-8.0]; *P* = .005) compared with those with higher educational attainment (Figure 1 and eTable 4 in Supplement 1). Black beneficiaries also enrolled in plans with a higher average number of dental benefits (IRR, 1.2 [95% CI, 1.1-1.4]; *P* < .001) compared with White beneficiaries (eTable 7 in Supplement 1).

#### Vision Benefits

**Figure 2.**
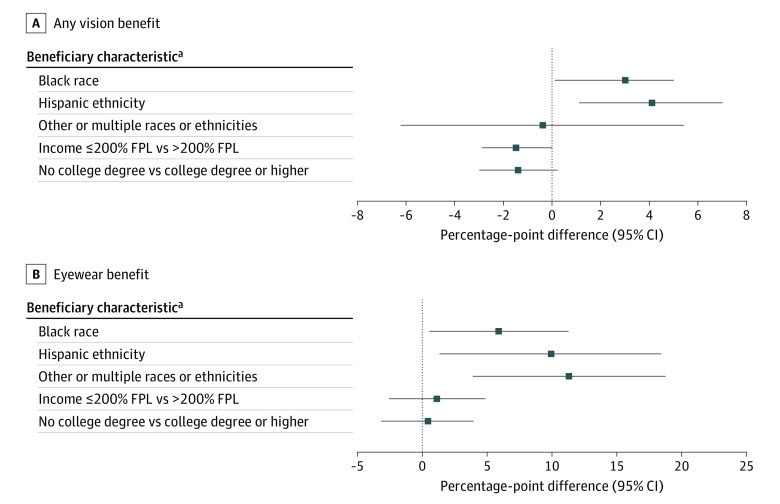
Average Adjusted Percentage-Point Difference in Enrollment Among Beneficiaries by Vision Benefits Adjusted marginal effects can be interpreted as adjusted percentage-point difference in enrollment and were derived from mixed-effects multivariable logistic regression models that included county-level random effect, beneficiary race and ethnicity, socioeconomic and demographic characteristics, rural-urban residence, health status, chronic illness burden, functional limitations, and plan features. FPL indicates federal poverty level. ^a^White race, higher income, and higher educational attainment served as the comparators.

Black beneficiaries and Hispanic beneficiaries were more likely to enroll in plans with an eye benefit (3.0 percentage-point difference [95% CI, 1.0-5.0]; *P* = .004; and 4.1 percentage-point difference [95% CI, 1.1-7.0]; *P* = .006) as well as in a plan with an eyewear benefit (6.0 percentage-point difference [95% CI, 0.6-11.5]; *P* = .03; and 10.1 percentage-point difference [95% CI, 1.4-18.7]; *P* = .02) compared with White beneficiaries. Individuals of other or multiple races or ethnicities were also more likely to enroll in plans with an eyewear benefit (11.5 percentage-point difference [95% CI, 4.0-19.0]; *P* = .003) compared with White beneficiaries (Figure 2 and eTable 5 in Supplement 1). The average number of eye benefits was not statistically significantly different across beneficiary characteristics (eTable 7 in Supplement 1).

#### Hearing Benefits

**Figure 3.**
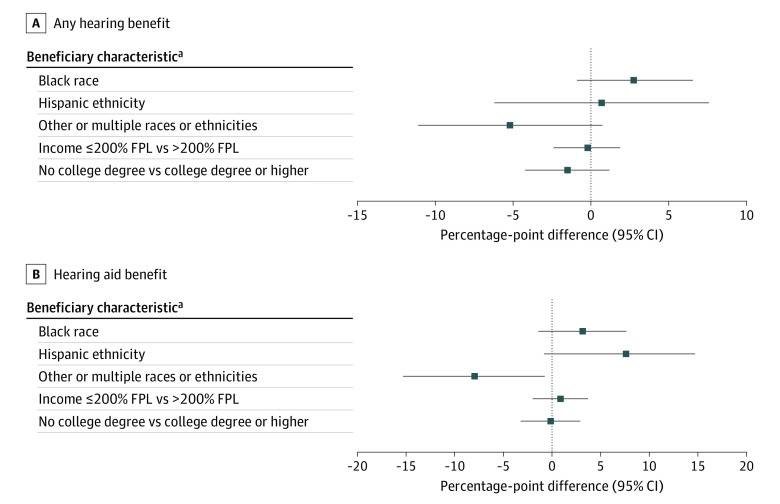
Average Adjusted Percentage-Point Difference in Enrollment Among Beneficiaries by Hearing Benefits Adjusted marginal effects can be interpreted as adjusted percentage-point difference in enrollment and were derived from mixed-effects multivariable logistic regression models that included county-level random effect, beneficiary race and ethnicity, socioeconomic and demographic characteristics, rural-urban residence, health status, chronic illness burden, functional limitations, and plan features. FPL indicates federal poverty level. ^a^White race, higher income, and higher educational attainment served as the comparators.

Beneficiaries of other or multiple races or ethnicities were less likely to enroll in plans with a hearing aid benefit (−7.9 percentage-point difference [95% CI, −15.2 to −0.7]; *P* = .03) compared with White beneficiaries (Figure 3 and eTable 6 in Supplement 1). In addition, these beneficiaries were enrolled in plans with a lower number of hearing benefits (IRR, 0.9 [95% CI, 0.8 to 0.9]; *P* = .02) (eTable 7 in Supplement 1).

### Sensitivity Analysis

Key differences from the primary analysis that were observed in the sensitivity analysis were as follows. In addition to race and ethnicity, beneficiaries without a college degree (vs higher educational attainment) were more likely to enroll in a plan with any dental benefit. Whereas Hispanic ethnicity was associated with enrollment in a plan with any eye benefit, a difference between Black and White beneficiaries was no longer observed. We additionally observed that health risks such as having more than 2 chronic conditions (vs none) or fair to poor health status (vs good to excellent) were associated with an increased likelihood of enrolling in a plan offering an eyewear benefit (eAppendix in Supplement 1).

## Discussion

Using nationally representative data on MA beneficiaries, we found that traditionally underserved beneficiaries (eg, of Black race or Hispanic ethnicity, with lower income, or with lower educational attainment) were more likely to enroll in plans that offer any or a comprehensive dental benefit. We also found that racial and ethnic minority group beneficiaries were more likely to enroll in plans with a vision benefit (particularly an eyewear benefit), and Hispanic beneficiaries were more likely to enroll in plans with a hearing aid benefit. In addition, beneficiaries with multiple chronic conditions or with fair to poor health status preferentially enrolled in plans with an eyewear benefit. Our findings align with an earlier study^28^ in which researchers reported higher enrollment of low-income, lower educational attainment, and Black or Hispanic beneficiaries in MA plans with any dental, vision, or hearing benefit. By examining the generosity of these benefits and controlling for beneficiary and plan characteristics, our study adds to the literature on MA plan selection decisions. Our findings of an association of these benefits with health characteristics were similar to studies by Feldman et al^29^ and Atherly et al^21^ nearly 20 years ago. Both studies observed unfavorable risk selection with vision benefits and none with hearing benefits, and Atherly et al^21^ found no risk selection by dental benefits.

Our findings suggest a reason for an increasing enrollment of traditionally underserved beneficiaries in MA plans,^2,30^ lending support for earlier speculation that MA supplementary benefits offered at no or a low additional premium are particularly attractive to low-income and racial and ethnic minority beneficiaries because they must buy standalone plans for these benefits if they choose TM.^13,14,31,32,33^ Findings from the Commonwealth Fund 2022 biennial survey showed that nearly a quarter of MA enrollees cite additional benefits as the main reason for choosing MA over TM, and this percentage was higher among lower-income compared with higher-income beneficiaries.^34^ Reid et al^26^ studied the role of MA plan attributes, such as costs, quality, and benefits, in beneficiary enrollment decisions. Although they did not discern the association of dental, vision, and hearing benefits separately, they found that these benefits, together with coverage type and cost sharing, explained almost 14% of the variation in plan choices. Because different factors are associated with plan choices among different beneficiaries,^26,27^ our results suggest that dental and vision benefits may explain a larger share of variation in plan choices across beneficiaries by race and ethnicity, income, or educational attainment.

Although our study reports on the characteristics of beneficiaries who are likely attracted to dental, vision, or hearing benefits, whether such preferential enrollment improves beneficiary access, outcomes, or quality of care is a critical gap in the literature. In contrast to the standard economic theory that favors more choices for consumers, research suggests that some Medicare beneficiaries have difficulty processing the complex information in MA due to expanded plan choices and heterogeneity, which deters their ability to match plan benefits to their health and financial needs.^22,33,35,36^ Across an average of 43 plans,^37^ beneficiaries must compare premiums, deductibles, cost sharing, out-of-pocket maximums, provider networks, prescription drug formularies, and supplemental benefits including dental, vision, or hearing.^22,33^ To facilitate such comparisons, efforts to improve transparency and decision-making have been suggested, such as coverage and costs associated with dental, vision, and hearing benefits detailed on the Medicare.gov website.^38^ Black and low-income beneficiaries commonly rely on plan-level marketing, which is concerning because of increasingly misleading marketing in MA.^34^ With prevailing information asymmetries in MA similar to other markets (eg, real estate) and complicated health plan choices, future research should investigate whether enrollment patterns or beneficiary plan choices are indeed beneficial to beneficiaries.

Evidence on benefit preferences of MA beneficiaries is particularly salient in the context of current policies aimed at reducing federal payments to MA plans, which may have consequences for funding of supplemental benefits, including dental, vision, and hearing.^13,14,15^ However, prior research suggests that in the current MA market, these consequences may be modest. Chernew et al^39^ reported that a $1000 decrease in benchmarks would lead to an approximately $60 increase in annual premiums and an approximately $27 increase in annual deductibles; in addition, the likelihood of offering benefits would decrease by less than 5 percentage points, with the greatest consequences on dental, vision, and hearing benefits. Schwartz et al^40^ also reported that the rate of MA enrollment growth did not decrease in response to reduced MA plan payments by the Affordable Care Act, suggesting that perhaps the benefits highly valued by beneficiaries were not reduced. However, this may be an artifact of the concurrent introduction of the quality bonus program. Along with payment cuts, the CMS plans to link plan payments with health equity through an adjustment of star ratings beginning in 2027 using the Health Equity Index.^16^ Although this policy may promote equity in Medicare, it may also incentivize MA plans to strategically reallocate their rebate dollars to alter supplemental benefits to attract or deter certain populations and game the positive intent of the Health Equity Index on promoting equity. The newly granted flexibilities by the CMS in the types of supplemental benefits that plans may offer^6^ could facilitate such reallocations. Although the response of MA plans to these policies is yet to be seen, tracking MA benefit offerings may help to ensure that the new policies do not inadvertently erode benefits, such as dental and vision, that are valued (whether appropriately or not) by marginalized Medicare beneficiaries.

### Limitations

Several limitations of our analyses must be noted. First, although we adjusted for plan characteristics that may influence plan enrollment decisions, we cannot causally attribute the enrollment patterns observed to dental, vision, or hearing benefits through this cross-sectional study. Second, the generosity of dental, vision, and hearing benefits can vary on several dimensions beyond those considered in our study, such as deductibles or annual plan maximums. Third, inclusion of general enrollment MA plan enrollees limits the generalizability of these findings to other beneficiaries. Fourth, an MCBS response rate of less than 100% may lead to selection bias. Fifth, the external validity of our study is limited by the MCBS sampling design methodology, which includes only approximately 10% of beneficiaries from facilities and has low county-level representation nationally (ie, beneficiaries from 138 counties in 2018, 135 in 2019, and 133 in 2020).^41^ Sixth, we could not examine enrollment patterns by standalone supplemental plans because of low enrollment, but we included these beneficiaries in a sensitivity analysis. Finally, we included data from survey years for which plan selection did not occur during the COVID-19 pandemic.

## Conclusions

The findings of this cross-sectional study suggest that traditionally underserved Medicare beneficiaries are more likely to enroll in MA plans with dental and vision supplemental benefits. Any benefit redesigns by MA plans could drive changes in enrollment patterns. The CMS is implementing major reforms to improve care and equity in MA, such as the Health Equity Index, MA payment cuts, and increased flexibility in supplemental benefit offerings. Information about benefit preferences of beneficiaries may inform CMS regulation of benefits and enable MA plans to better meet the needs of underserved beneficiaries.

## Notes

Supplement 1. **eFigure 1.** Study Sample Selection Flowchart by Year **eTable 1.** Sample Characteristics of Medicare Advantage (MA) Beneficiaries Enrolled in General Enrollment MA Plans, 2018 to 2020 Medicare Current Beneficiary Survey **eTable 2.** Sample Characteristics of Medicare Advantage (MA) Beneficiaries Enrolled in General Enrollment MA Plans But Not in Dental, Vision, or Hearing Standalone Plans, 2018 to 2020 Medicare Current Beneficiary Survey **eTable 3.** Beneficiary Characteristics by Enrollment in a Plan With and Without a Dental, Vision, or Hearing Benefit, 2018 to 2020 Medicare Current Beneficiary Survey **eTable 4.** Adjusted Percentage-Point Difference in Beneficiary Enrollment by Dental Benefits, 2018 to 2020 Medicare Current Beneficiary Survey **eTable 5.** Adjusted Percentage-Point Difference in Beneficiary Enrollment by Vision Benefits, 2018 to 2020 Medicare Current Beneficiary Survey **eTable 6.** Adjusted Percentage-Point Difference in Beneficiary Enrollment by Hearing Benefits, 2018 to 2020 Medicare Current Beneficiary Survey **eTable 7.** Association Between Beneficiary Characteristics and Number of Dental, Vision, and Hearing Benefits, 2018 to 2020 Medicare Current Beneficiary Survey **eAppendix.** Sensitivity Analysis

Supplement 2.
Data Sharing Statement

## References

[1] Biniek JF, Freed M, Damico A, Neuman T. Half of all eligible Medicare beneficiaries are now enrolled in private Medicare Advantage plans. KFF. May 1, 2023. Accessed May 5, 2023. https://www.kff.org/policy-watch/half-of-all-eligible-medicare-beneficiaries-are-now-enrolled-in-private-medicare-advantage-plans/

[2] Meyers DJ, Mor V, Rahman M, Trivedi AN. Growth in Medicare Advantage greatest among Black and Hispanic enrollees. Health Aff (Millwood). 2021;40(6):945-950. doi:10.1377/hlthaff.2021.00118 34097525 PMC8297509

[3] Findlay S, Jacobson G, Cicchiello A. Medicare Advantage: a policy primer. The Commonwealth Fund. 2022. Accessed December 1, 2022. doi:10.26099/69fq-dy83

[4] MedPAC. The Medicare Advantage program: status report and mandated report on dual-eligible special needs plans. 2022. Accessed January 4, 2023. https://www.medpac.gov/document/chapter-12-the-medicare-advantage-program-status-report-and-mandated-report-on-dual-eligible-special-needs-plans-march-2022-report/

[5] MedPAC. Medicare Advantage Program Payment System. November 2021. Accessed December 5, 2022. https://www.medpac.gov/wp-content/uploads/2021/11/medpac_payment_basics_21_ma_final_sec.pdf

[6] Kornfield T, Kazan M, Frieder M, Duddy-Tenbrunsel R, Donthi S, Fix A. Medicare Advantage plans offering expanded supplemental benefits: a look at availability and enrollment. The Commonwealth Fund. 2021. Accessed November 5, 2022. doi:10.26099/345k-kc32

[7] Freed M, Ochieng N, Sroczynski N, Damico A, Amin K. Medicare and dental coverage: a closer look. KFF. 2021. Accessed August 13, 2022. https://www.kff.org/medicare/issue-brief/medicare-and-dental-coverage-a-closer-look/

[8] Congressional Budget Office. Letter to the Honorable Frank Pallone Jr: budgetary effects of H.R. 3, the Elijah E. Cummings Lower Drug Costs Now Act. December 10, 2019. Accessed May 18, 2023. https://www.cbo.gov/system/files/2019-12/hr3_complete.pdf

[9] Centers for Disease Control and Prevention Division of Oral Health. Older adult oral health. Updated May 5, 2021. Accessed December 14, 2022. https://www.cdc.gov/oralhealth/basics/adult-oral-health/adult_older.htm#:~:text=Nearly%20all%20adults%20(96%25),5%20have%20untreated%20tooth%20decay.&text=Gum%20disease.,or%20older%20have%20gum%20disease

[10] Freed M, Cubanski J, Sroczynski N, Ochieng N, Neuman T. Dental, hearing, and vision costs and coverage among Medicare beneficiaries in traditional Medicare and Medicare Advantage. KFF. 2021. Accessed May 6, 2022. https://www.kff.org/health-costs/issue-brief/dental-hearing-and-vision-costs-and-coverage-among-medicare-beneficiaries-in-traditional-medicare-and-medicare-advantage/

[11] Trish E, Valdez S, Ginsburg PB, Randall S, Lieberman SM. Substantial growth in Medicare Advantage and implications for reform. Health Aff (Millwood). 2023;42(2):246-251. doi:10.1377/hlthaff.2022.00668 36745825

[12] Centers for Medicare & Medicaid Services. Serving vulnerable and underserved populations. September 21, 2021. Accessed September 12, 2023. https://www.hhs.gov/guidance/sites/default/files/hhs-guidance-documents/006_Serving_Vulnerable_and_Underserved_Populations.pdf

[13] The Commonwealth Fund. Study: low-income and minority populations use Medicare Advantage plans. September 20, 2005. Accessed May 29, 2021. https://www.commonwealthfund.org/publications/newsletter-article/study-low-income-and-minority-populations-use-medicare-advantage

[14] Thorpe KE, Atherly A. Medicare+Choice: current role and near-term prospects. Health Aff (Millwood). 2002;21(Suppl Web Exclusives):W242-W252. doi:10.1377/hlthaff.W2.242 12703580

[15] Song A, Creighton S, Duddy-Tenbrunsel R, Godoy S. Proposed MA plan payment changes may impact premiums and benefits. Avalere. 2023. Accessed September 12, 2023. https://avalere.com/insights/proposed-ma-plan-payment-changes-may-impact-premiums-and-benefits

[16] Centers for Medicare & Medicaid Services. 2024 Medicare Advantage and Part D Final Rule (CMS-4201-F). April 4, 2023. Accessed August 12, 2023. https://www.cms.gov/newsroom/fact-sheets/2024-medicare-advantage-and-part-d-final-rule-cms-4201-f

[17] Meyers DJ, Durfey SNM, Gadbois EA, Thomas KS. Early adoption of new supplemental benefits by Medicare Advantage plans. JAMA. 2019;321(22):2238-2240. doi:10.1001/jama.2019.4709 31184727 PMC6563562

[18] Centers for Medicare & Medicaid Services. 2020 MCBS methodology report. 2020. Accessed June 30, 2023. https://www.cms.gov/files/document/2020-mcbs-methodology-report.pdf

[19] Crook HL, Zhao AT, Saunders RS. Analysis of Medicare Advantage plans’ supplemental benefits and variation by county. JAMA Netw Open. 2021;4(6):e2114359. doi:10.1001/jamanetworkopen.2021.14359 34160609 PMC8223098

[20] Meyers DJ, Gadbois EA, Brazier J, Tucher E, Thomas KS. Medicare plans’ adoption of special supplemental benefits for the chronically ill for enrollees with social needs. JAMA Netw Open. 2020;3(5):e204690. doi:10.1001/jamanetworkopen.2020.4690 32396191 PMC7218486

[21] Atherly A, Dowd BE, Feldman R. The effect of benefits, premiums, and health risk on health plan choice in the Medicare program. Health Serv Res. 2004;39(4 Pt 1):847-864. doi:10.1111/j.1475-6773.2004.00261.x 15230931 PMC1361041

[22] McWilliams JM, Afendulis CC, McGuire TG, Landon BE. Complex Medicare advantage choices may overwhelm seniors—especially those with impaired decision making. Health Aff (Millwood). 2011;30(9):1786-1794. doi:10.1377/hlthaff.2011.0132 21852301 PMC3513347

[23] Johnston KJ, Hammond G, Meyers DJ, Joynt Maddox KE. Association of race and ethnicity and Medicare program type with ambulatory care access and quality measures. JAMA. 2021;326(7):628-636. doi:10.1001/jama.2021.10413 34402828 PMC8371568

[24] Madden JM, Bayapureddy S, Briesacher BA, . Affordability of medical care among Medicare enrollees. JAMA Health Forum. 2021;2(12):e214104. doi:10.1001/jamahealthforum.2021.4104 35977305 PMC8796945

[25] Park S, Werner RM, Coe NB. Racial and ethnic disparities in access to and enrollment in high-quality Medicare Advantage plans. Health Serv Res. 2023;58(2):303-313. doi:10.1111/1475-6773.13977 35342936 PMC10012240

[26] Reid RO, Deb P, Howell BL, Conway PH, Shrank WH. The roles of cost and quality information in Medicare Advantage plan enrollment decisions: an observational study. J Gen Intern Med. 2016;31(2):234-241. doi:10.1007/s11606-015-3467-3 26282952 PMC4720649

[27] Reid RO, Deb P, Howell BL, Shrank WH. Association between Medicare Advantage plan star ratings and enrollment. JAMA. 2013;309(3):267-274. doi:10.1001/jama.2012.173925 23321765

[28] Willink A. The high coverage of dental, vision, and hearing benefits among Medicare Advantage enrollees. Inquiry. 2019;56:46958019861554. 31271082 10.1177/0046958019861554 PMC6611012

[29] Feldman R, Dowd B, Wrobel M. Risk selection and benefits in the Medicare+Choice program. Health Care Financ Rev. 2003;25(1):23-36. 14997691 PMC4194834

[30] Murphy-Barron CPB, Ferro C, Emery M. Comparing the demographics of enrollees in Medicare Advantage and fee-for-service Medicare. 2020. Accessed May 20, 2021. https://bettermedicarealliance.org/wp-content/uploads/2020/10/Comparing-the-Demographics-of-Enrollees-in-Medicare-Advantage-and-Fee-for-Service-Medicare-202010141.pdf

[31] Berenson RA, Dowd BE. Medicare advantage plans at a crossroads—yet again. Health Aff (Millwood). 2009;28(1):w29-w40. 19029151 10.1377/hlthaff.28.1.w29

[32] Pope C. Supplemental benefits under Medicare Advantage. *Health Affairs Blog*. January 21, 2016. Accessed May 20, 2021. https://www.healthaffairs.org/content/forefront/supplemental-benefits-under-medicare-advantage

[33] Rivera-Hernandez M, Blackwood KL, Moody KA, Trivedi AN. Plan switching and stickiness in Medicare Advantage: a qualitative interview with Medicare Advantage beneficiaries. Med Care Res Rev. 2021;78(6):693-702. doi:10.1177/1077558720944284 32744130 PMC7903586

[34] Leonard F, Jacobson G, Haynes LA, Collins SR. Traditional Medicare or Medicare Advantage: how older Americans choose and why. The Commonwealth Fund. 2022. Accessed May 20, 2023. doi:10.26099/2rfq-z770

[35] Jacobson G, Swoope C, Perry M, Slosar MC. How are seniors choosing and changing health insurance plans? KFF. May 13, 2014. Accessed August 20, 2023. https://www.kff.org/medicare/report/how-are-seniors-choosing-and-changing-health-insurance-plans/

[36] Afendulis CC, Sinaiko AD, Frank RG. Dominated choices and Medicare Advantage enrollment. J Econ Behav Organ. 2015;119:72-83. doi:10.1016/j.jebo.2015.07.009 26339108 PMC4553698

[37] Freed M, Fuglesten Biniek J, Damico A, Neuman T. Medicare Advantage 2023 spotlight: first look. Kaiser Family Foundation. November 10, 2022. Accessed June 20, 2023. https://www.kff.org/medicare/issue-brief/medicare-advantage-2023-spotlight-first-look/

[38] Grabert LM. Medicare must provide additional cost and access information to enhance decision making around trade offs between Medicare Advantage and Medigap. Inquiry. 2022;59:469580221094469. 35506691 10.1177/00469580221094469 PMC9073103

[39] Chernew ME, Miller K, Petrin A, Town RJ. Reducing Medicare Advantage benchmarks will decrease plan generosity, but those effects will likely be modest. Health Aff (Millwood). 2023;42(4):479-487. doi:10.1377/hlthaff.2022.01031 36947715

[40] Schwartz AL, Kim S, Navathe AS, Gupta A. Growth of Medicare Advantage after plan payment reductions. JAMA Health Forum. 2023;4(6):e231744. doi:10.1001/jamahealthforum.2023.1744 37354538 PMC10290750

[41] Centers for Medicare & Medicaid Services. 2019 MCBS methodology report. 2019. Accessed May 10, 2022. https://www.cms.gov/files/document/2019-mcbs-methodology-report.pdf

