# Supplementary material for: Enrollment Patterns of Medicare Advantage Beneficiaries by Dental, Vision, and Hearing Benefits
Source: JAMA Health Forum. 2024 Jan 12;5(1):e234936. doi: 10.1001/jamahealthforum.2023.4936 (PMC10787318; doi:10.1001/jamahealthforum.2023.4936)
Supplement: Supplement 1. — eFigure 1. Study Sample Selection Flowchart by Year eTable 1. Sample Characteristics of Medicare Advantage (MA) Beneficiaries Enrolled in General Enrollment MA Plans, 2018 to 2020 Medicare Current Beneficiary Survey eTable 2. Sample Characteristics of Medicare Advantage (MA) Beneficiaries Enrolled in General Enrollment MA Plans But Not in Dental, Vision, or Hearing Standalone Plans, 2018 to 2020 Medicare Current Beneficiary Survey eTable 3. Beneficiary Characteristics by Enrollment in a Plan With and Without a Dental, Vision, or Hearing Benefit, 2018 to 2020 Medicare Current Beneficiary Survey eTable 4. Adjusted Percentage-Point Difference in Beneficiary Enrollment by Dental Benefits, 2018 to 2020 Medicare Current Beneficiary Survey eTable 5. Adjusted Percentage-Point Difference in Beneficiary Enrollment by Vision Benefits, 2018 to 2020 Medicare Current Beneficiary Survey eTable 6. Adjusted Percentage-Point Difference in Beneficiary Enrollment by Hearing Benefits, 2018 to 2020 Medicare Current Beneficiary Survey eTable 7. Association Between Beneficiary Characteristics and Number of Dental, Vision, and Hearing Benefits, 2018 to 2020 Medicare Current Beneficiary Survey eAppendix. Sensitivity Analysis [file jamahealthforum-e234936-s001.pdf]

Supplementary Online Content

Gupta A, Silver D, Meyers DJ, Murray G, Glied S, Pagán JA. Enrollment patterns of Medicare Advantage beneficiaries by dental, vision, and hearing benefits. *JAMA Health Forum*. 2024;5(1):e234936. doi:10.1001/jamahealthforum.2023.4936

eFigure 1. Study Sample Selection Flowchart by Year

eTable 1. Sample Characteristics of Medicare Advantage (MA) Beneficiaries Enrolled in General Enrollment MA Plans, 2018 to 2020 Medicare Current Beneficiary Survey

eTable 2. Sample Characteristics of Medicare Advantage (MA) Beneficiaries Enrolled in General Enrollment MA Plans But Not in Dental, Vision, or Hearing Standalone Plans, 2018 to 2020 Medicare Current Beneficiary Survey

eTable 3. Beneficiary Characteristics by Enrollment in a Plan With and Without a Dental, Vision, or Hearing Benefit, 2018 to 2020 Medicare Current Beneficiary Survey

eTable 4. Adjusted Percentage-Point Difference in Beneficiary Enrollment by Dental Benefits, 2018 to 2020 Medicare Current Beneficiary Survey

eTable 5. Adjusted Percentage-Point Difference in Beneficiary Enrollment by Vision Benefits, 2018 to 2020 Medicare Current Beneficiary Survey

eTable 6. Adjusted Percentage-Point Difference in Beneficiary Enrollment by Hearing Benefits, 2018 to 2020 Medicare Current Beneficiary Survey

eTable 7. Association Between Beneficiary Characteristics and Number of Dental, Vision, and Hearing Benefits, 2018 to 2020 Medicare Current Beneficiary Survey

eAppendix. Sensitivity Analysis

This supplementary material has been provided by the authors to give readers additional information about their work.

© 2023 Gupta A et al. *JAMA Health Forum*.

eFigure 1. Study Sample Selection Flowchart by Year

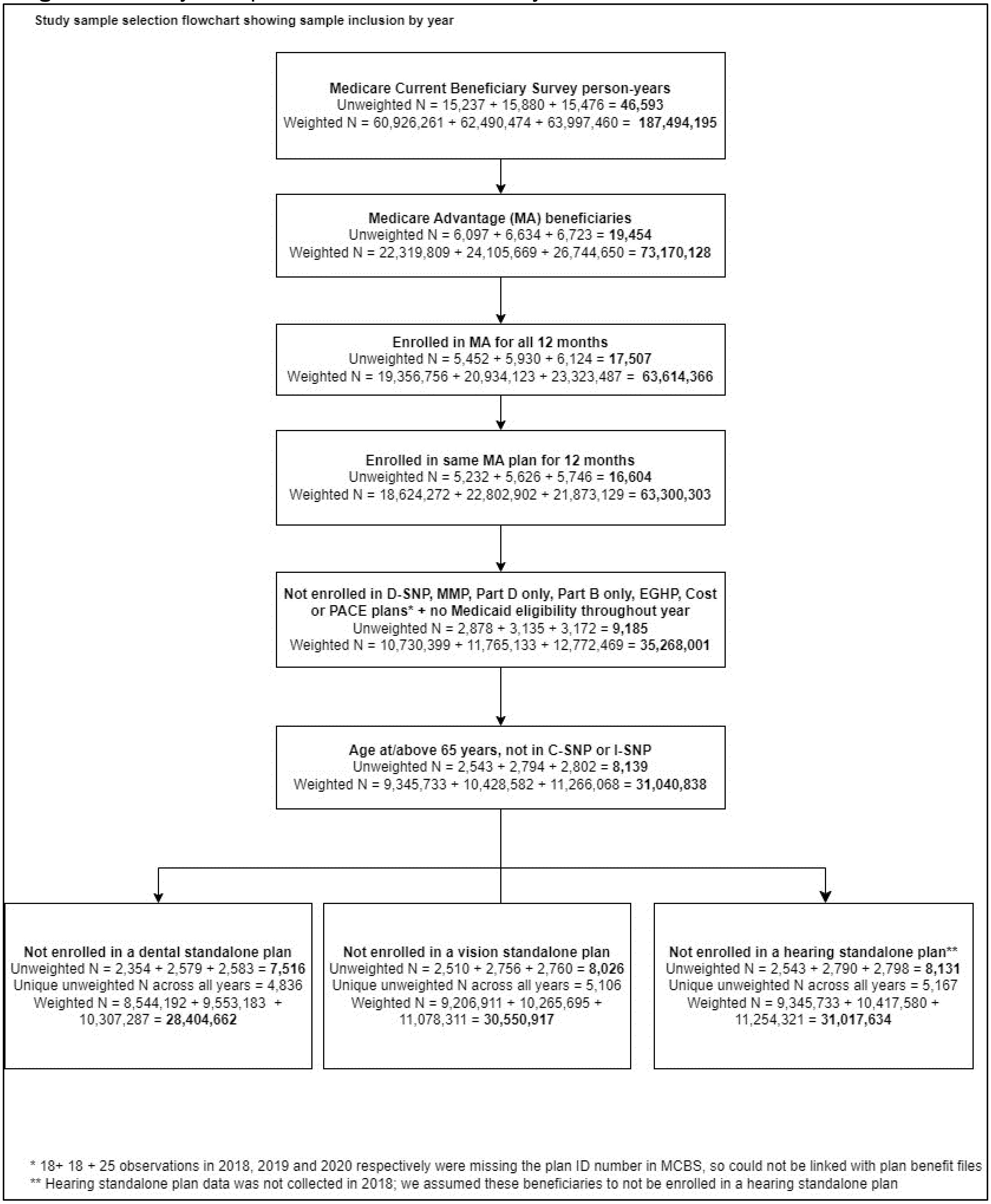

© 2023 Gupta A et al. *JAMA Health Forum*.

eTable 1. Sample Characteristics of Medicare Advantage (MA) Beneficiaries Enrolled in General Enrollment MA Plans, 2018 to 2020 Medicare Current Beneficiary Survey

	**Weighted percentage (%)**	**(95% CI)**
	N=8,139	
Race/ethnicity		
Black	9.8	8.4 to 11.3
Hispanic	2.0	1.6 to 2.7
Other/multiple	4.2	3.4 to 5.3
White	83.9	81.9 to 85.7
Income level		
≤200% FPL	40.7	38.6 to 42.8
>200% FPL	59.3	57.2 to 61.4
Education[a]		
Not completed college	67.1	64.7 to 69.3
College degree or higher	32.9	30.1 to 35.3
Age		
65-74	54.9	53.0 to 56.7
75-84	32.9	31.5 to 34.3
≥85	12.2	11.3 to 13.1
Sex		
Men	45.0	43.2 to 46.8
Women	54.9	53.2 to 56.8
Marital status[b]		
Married	54.8	53.0 to 56.6
Widow/divorced/separated/unmarried	45.1	43.3 to 46.9
Health status[c]		
Fair/poor	14.4	13.5 to 15.4
Good/excellent	85.5	84.5 to 86.5
Functional limitation[d]		
None	66.9	65.3 to 68.6
Only IADL	11.4	10.5 to 12.4
1-2 ADL	15.6	14.6 to 16.7
3-6 ADL	5.9	5.3 to 6.7
Chronic illness burden[e]		
No	32.1	30.3 to 33.9
1	35.2	33.4 to 36.9
2	18.8	17.6 to 20.0
>2	12.8	12.5 to 15.3
Dental standalone plan		
Yes	8.5	7.2 to 10.0
Vision standalone plan		
Yes	1.6	1.2 to 2.1
Hearing standalone plan[f]		
Yes	0.1	0.03 to 0.3
Any dental benefit[g]		
Yes	82.6	78.7 to 85.9
Any mandatory dental benefit[g]		
Yes	68.5	63.9 to 72.7
Any mandatory comprehensive dental benefit[g]		
Yes	42.1	39.2 to 45.1
Number of mandatory dental benefits (mean)[g]	4.1	3.8 to 4.3
Any eye benefit[h]		
Yes	98.5	97.4 to 99.1

© 2023 Gupta A et al. *JAMA Health Forum*.

	Weighted percentage (%)	(95% CI)
	N=8,139	
Any mandatory eye benefit[h]		
Yes	95.7	94.2 to 96.9
Any mandatory eye wear benefit[h]		
Yes	74.9	70.0 to 79.1
Number of mandatory eye benefits (mean)[h]	3.2	3.1 to 3.4
Any hearing benefit[i]		
Yes	93.3	91.0 to 95.0
Any mandatory hearing benefit[i]		
Yes	86.2	82.6 to 89.2
Any mandatory hearing aid benefit[i]		
Yes	78.4	74.8 to 81.6
Number of mandatory hearing benefits (mean)[i]	2.1	2.1 to 2.2

Abbreviations: FPL: Federal Poverty Level; ADL: Activities of Daily Living; IADL: Instrumental Activities of Daily Living
[a]Education missing for 1.9%; [b]Marital status missing for 0.1%; [c]Health status missing for 4.7%; [d]Functional limitation missing for 4.5%; [e]Chronic condition missing for 4.4%; [f]Hearing standalone plan data was not collected in MCBS 2018; [g]Among those without dental standalone plan (N=7,516); [h]Among those without vision standalone plan (N=8,026); [i]Among those without hearing standalone plan

© 2023 Gupta A et al. *JAMA Health Forum*.

eTable 2. Sample Characteristics of Medicare Advantage (MA) Beneficiaries Enrolled in General Enrollment MA Plans But Not in Dental, Vision, or Hearing Standalone Plans, 2018 to 2020 Medicare Current Beneficiary Survey

	DENTAL N= 7,516		VISION N=8,026		HEARING N=8,131	
	**Weighted percentage (%)**	**(95% CI)**	**Weighted percentage (%)**	**(95% CI)**	**Weighted percentage (%)**	**(95% CI)**
Race/ethnicity						
Black	10.1	8.6, 11.7	9.8	8.4, 11.3	9.8	8.4, 11.3
Hispanic	2.1	1.6, 2.7	2.1	1.6, 2.7	2.0	1.6, 2.7
Other	4.4	3.5, 5.5	4.3	3.5, 5.4	4.2	3.4, 5.3
White	83.4	81.3, 85.3	83.7	81.7, 85.6	83.9	81.9, 85.7
Income level						
≤200% FPL	42.9	40.8, 45.0	41.1	39.0, 43.2	40.7	38.6, 42.8
>200% FPL	57.1	54.9, 59.1	58.9	56.7, 60.9	59.3	57.2, 61.4
Education						
Not completed college	68.5	66.2, 70.6	67.3	64.9, 69.6	67.1	64.7, 69.3
College degree or higher	31.5	29.3, 33.7	32.6	30.3, 35.0	32.9	30.1, 35.3
Age						
65-74	53.7	51.7, 55.6	54.5	52.7, 56.3	54.9	53.0, 56.7
75-84	33.6	32.1, 35.2	33.2	31.7, 34.6	32.9	31.5, 34.3
≥85	12.7	11.7, 13.7	12.3	11.4, 13.2	12.2	11.3, 13.1
Sex						
Men	45.3	43.5, 47.1	44.8	43.0, 46.7	44.9	43.2, 46.8
Women	54.7	52.8, 56.5	55.2	53.3, 56.9	55.0	53.2, 56.8
Marital status						
Married	53.4	51.6, 55.3	54.4	52.6, 56.2	54.8	53.1, 56.6
Not married	46.6	44.7, 48.4	45.6	43.7, 47.4	45.2	43.3, 46.9
Rural Urban residence						
Urban	88.2	85.6, 90.4	88.6	86.0, 90.8	88.6	86.1, 90.7
Rural	11.7	9.5, 14.4	11.3	9.2, 13.9	11.3	9.2, 13.9
Health status						
Fair/poor	14.8	13.8, 15.8	14.6	13.6, 15.7	14.4	13.5, 15.4
Good/excellent	85.2	84.2, 86.2	85.4	84.3, 86.3	85.5	84.5, 86.5
Functional limitation						
None	66.4	64.7, 67.9	66.7	65.0, 68.4	66.9	65.3, 68.6
Only IADL	11.5	10.5, 12.7	11.5	10.5,12.5	11.4	10.5, 12.4
1-2 ADL	16.1	15.1, 17.2	15.8	14.8,16.8	15.6	14.6, 16.7

© 2023 Gupta A et al. *JAMA Health Forum*.

	DENTAL<br>N= 7,516		VISION<br>N=8,026		HEARING<br>N=8,131	
	**Weighted percentage (%)**	**(95% CI)**	**Weighted percentage (%)**	**(95% CI)**	**Weighted percentage (%)**	**(95% CI)**
3-6 ADL	5.9	5.3, 6.7	5.9	5.3, 6.7	5.9	5.3, 6.7
Chronic illness burden						
No	32.3	30.5, 34.1	32.1	30.3, 33.9	32.1	30.3, 33.9
1	34.6	32.7,36.5	35.3	33.5, 37.1	35.2	33.4, 36.9
2	19.1	17.8, 20.3	18.7	17.5, 19.8	18.8	17.6, 20.0
>2	14.1	12.6, 15.6	13.9	12.6, 15.5	13.8	12.5, 15.3
Monthly plan premium (C+D)						
Zero dollar	67.5	63.3, 71.4	67.1	62.9, 71,1	67.1	62.8, 71.1
≤$51.00	20.9	18.5, 23.6	21.1	18.7,23.7	21.1	18.7, 23.7
>51.00	11.6	8.8, 15.1	11.7	9.0, 15.2	11.7	9.0, 15.2
Annual Plan OOP max[a]						
<$3,500	25.9	21.1, 31.5	25.7	20.7, 31.3	25.6	20.6, 31.3
$3,501 - $4,950	25.4	22.1, 29.3	25.8	22.3, 29.7	25.9	22.3, 29.8
$4,951 - $6,700	19.2	16.1, 22.6	19.4	16.4, 22.7	19.4	16.4, 22.9
>$6,700	29.3	25.7, 33.2	29.1	25.4, 33.1	29.0	25.4, 32.9
Plan star rating						
<4	21.8	19.2, 24.6	21.6	19.0, 24.5	21.8	19.1, 24.7
≥4	78.2	75.4, 80.7	78.3	75.4, 80.9	78.2	75.2, 80.9
Type of coverage						
HMO	70.9	67.8, 73.8	70.9	68.0, 73.7	70.9	68.0, 73.7
PPO/PFFS	29.1	26.2, 32.1	29.0	26.3, 31.9	29.1	26.3, 31.9
At least one mandatory dental/vision/hearing benefit	68.5	63.9, 72.7	95.7	94.2, 96.9	85.4	81.7, 88.4
At least one mandatory dental comprehensive/eye wear/ hearing aid benefit	42.0	39.2, 45.0	74.8	70.0, 79.1	74.7	70.5, 78.6
Number of mandatory benefits (mean)	4.1	3.8, 4.3	3.2	3.1, 3.4	2.1	2.0, 2.2

Abbreviations: FPL: Federal Poverty Level; ADL: Activities of Daily Living; IADL: Instrumental Activities of Daily Living; OOP: Out-of-pocket; HMO: Health Maintenance Organization; PPO/PFFS: Preferred Provider Organization/Private Fee-for-Service; Weighted percentages represent the column percentages; [a]Plan out-of-pocket maximum missing for 1.4%

© 2023 Gupta A et al. *JAMA Health Forum*.

eTable 3. Beneficiary Characteristics by Enrollment in a Plan With and Without a Dental, Vision, or Hearing Benefit, 2018 to 2020 Medicare Current Beneficiary Survey

	**DENTAL N= 7,516 Weighted % (95% CI)**		**VISION N=8,026 Weighted % (95% CI)**		**HEARING N=8,131 Weighted % (95% CI)**	
**Enrolled in a MA plan→**	**with dental benefit**	**without dental benefit**	**with eye benefit**	**without eye benefit**	**with hearing benefit**	**without hearing benefit**
Overall	68.5 (63.9 to 72.8)	31.5 (27.2 to 36.1)	95.8 (94.2 to 96.9)	4.2 (3.0 to 5.8)	85.4 (81.7 to 88.4)	14.6 (11.5 to 18.3)
Race/ethnicity						
Black	77.1 (70.6 to 82.6)	22.8 (17.3 to 29.4)	96.7 (92.0 to 98.7)	3.3 (1.3 to 8.0)	88.2 (83.6 to 91.6)	11.8 (8.4 to 16.4)
Hispanic	67.7 (57.8 to 76.2)	32.3 (23.8 to 42.2)	99.3 (95.2 to 99.9)	0.7 (0.1 to 4.8)	88.0 (80.5 to 92.9)	12.0 (7.1 to 19.5)
Other/multiple	52.9 (40.9 to 64.4)	47.1%[a] (35.5 to 59.0)	97.3 (91.5 to 99.2)	2.6 (0.8 to 8.5)	77.6 (66.4 to 85.8)	22.4 (14.2 to 33.6)
White	68.3 (63.7 to 72.6)	31.7 (27.4 to 36.3)	95.5 (93.6 to 96.9)	4.5 (3.1 to 6.4)	85.4 (81.4 to 88.7)	14.6 (11.3 to 18.6)
Income level						
≤200% FPL	69.4 (64.7 to 73.7)	30.5 (26.2 to 35.2)	95.0 (93.0 to 96.4)	5.0 (3.5 to 7.0)	85.1 (81.3 to 88.2)	14.9 (11.8 to 18.7)
>200% FPL	67.8 (62.7 to 72.5)	32.2 (27.5 to 37.3)	96.3 (94.8 to 97.4)	3.7 (2.6 to 5.2)	85.6 (81.5 to 88.9)	14.4 (11.1 to 18.4)
Education[b]						
No college degree	70.4 (66.1 to 74.4)	29.5 (25.5 to 33.9)	95.3 (93.5 to 96.6)	4.7 (3.4 to 6.5)	85.9 (82.0 to 89.1)	14.1 (10.9 to 18.1)
≥College degree	64.2 (57.7 to 70.2)	35.8 (29.8 to 42.2)	96.9 (95.3 to 97.9)	3.1 (2.1 to 4.7)	84.5 (80.5 to 87.8)	15.5 (12.2 to 19.5)

© 2023 Gupta A et al. *JAMA Health Forum*.

	DENTAL<br>N= 7,516<br>Weighted % (95% CI)		VISION<br>N=8,026<br>Weighted % (95% CI)		HEARING<br>N=8,131<br>Weighted % (95% CI)	
**Enrolled in a MA plan→**	**with dental benefit**	**without dental benefit**	**with eye benefit**	**without eye benefit**	**with hearing benefit**	**without hearing benefit**
Age						
65-74	68.7<br>(63.9 to 73.1)	31.2%<br>(26.8 to 36.0)	95.9<br>(94.1 to 97.2)	4.0<br>(2.7 to 5.8)	85.3<br>(81.2 to 88.6)	14.7<br>11.4 to 18.8)
75-84	68.2<br>(62.9 to 73.0)	31.8<br>(27.0 to 37.1)	95.6<br>(93.9 to 96.9)	4.4<br>(3.1 to 6.2)	85.0<br>(80.9 to 88.3)	15.0<br>(11.6 to 19.0)
≥85	68.4<br>(62.9 to 73.4)	31.6<br>(26.6 to 37.0)	95.4<br>(93.7 to 96.6)	4.6<br>(3.4 to 6.2)	87.0<br>(82.7 to 90.3)	13.0<br>(9.6 to 17.3)
Sex						
Male	68.3<br>6(3.1 to 73.0)	31.7<br>(26.9 to 36.9)	95.5<br>(93.5 to 96.9)	4.4<br>(3.0 to 6.5)	84.8<br>(80.4 to 88.4)	15.2<br>11.6 to 19.6)
Female	68.7<br>(64.1 to 72.9)	31.3<br>(27.0 to 35.9)	95.9<br>(94.4 to 97.1)	4.1<br>(2.9 to 5.5)	85.9<br>(82.4 to 88.7)	14.1<br>(11.3 to 17.5)
Marital status[c]						
Married	68.4<br>(63.1 to 73.3)	31.6<br>(26.7 to 36.9)	95.9<br>(94.1 to 97.1)	4.1<br>(2.8 to 5.9)	85.8<br>(81.8 to 89.0)	14.2<br>(10.9 to 18.2)
Not married	68.6<br>(64.2 to 72.7)	31.4<br>(27.3 to 35.8)	95.7<br>(93.9 to 96.9)	4.4<br>(3.0 to 6.1)	84.9<br>(80.9 to 88.2)	15.1<br>(11.8 to 19.1)
Rural Urban residence						
Urban	67.7<br>(62.8 to 72.2)	32.3<br>(27.8 to 37.2)	96.3<br>(94.6 to 97.5)	3.7<br>2.5 to 5.4)	85.9<br>(82.1 to 89.1)	14.0<br>(10.8 to 17.9)
Rural	74.8<br>(66.8 to 81.4)	25.2<br>(18.6 to 33.1)	91.9<br>(85.8 to 95.5)	8.1<br>(4.5 to 14.2)	80.9<br>(71.5 to 87.8)	19.0<br>(12.2 to 28.4)
Health status[d]						
Fair/poor	69.7	30.2	95.9	4.1	86.7	13.3

© 2023 Gupta A et al. *JAMA Health Forum*.

	DENTAL<br>N= 7,516<br>Weighted % (95% CI)		VISION<br>N=8,026<br>Weighted % (95% CI)		HEARING<br>N=8,131<br>Weighted % (95% CI)	
**Enrolled in a MA plan→**	**with dental benefit**	**without dental benefit**	**with eye benefit**	**without eye benefit**	**with hearing benefit**	**without hearing benefit**
	(64.4 to 74.6)	(25.4 to 35.6)	(93.9 to 97.3)	(2.7 to 6.1)	(82.9 to 89.8)	(10.2 to 17.1)
Good/excellent	68.6<br>(63.9 to 72.9)	31.4<br>(27.0 to 36.1)	95.8<br>(93.9 to 97.1)	4.2<br>(2.9 to 6.1)	85.4<br>(81.4 to 88.6)	14.6<br>(11.4 to 18.5)
Functional limitation[e]						
None	68.2<br>(63.4 to 72.6)	31.8<br>(27.4 to 36.5)	96.1<br>94.3 to 97.4)	3.9<br>(2.6 to 5.7)	85.5<br>(81.4 to 88.9)	14.4<br>(11.1 to 18.6)
Only IADL	71.6<br>(66.0 to 76.5)	28.4<br>(23.5 to 33.9)	95.0<br>(92.5 to 96.7)	4.9<br>(3.2 to 7.5)	85.5<br>(81.3 to 88.8)	14.5<br>(11.1 to 18.6)
1-2 ADL	68.3<br>(63.0 to 73.3)	31.7<br>(26.8 to 36.9)	95.7<br>(93.7 to 97.2)	4.3<br>(2.9 to 6.3)	86.4<br>(83.3 to 89.1)	13.6<br>(10.9 to 16.6)
3-6 ADL	71.6<br>(64.5 to 77.8)	28.4<br>(22.2 to 35.5)	94.5<br>(91.6 to 96.5)	5.4<br>(3.5 to 8.3)	83.6<br>(77.2 to 88.4)	16.4<br>(11.6 to 22.7)
Chronic illness burden[f]						
No	68.0<br>(63.1 to 72.6)	31.9<br>(27.5 to 36.9)	96.2<br>94.2 to 97.6)	3.8<br>2.4 to 5.8)	86.4<br>(81.9 to 89.90	13.6<br>(10.1 to 18.1)
1	68.1<br>(62.3 to 73.4)	31.8<br>(26.6 to 37.7)	95.6<br>(93.9 to 96.8)	4.4<br>(3.2 to 6.1)	85.9<br>(82.1 to 88.9)	14.1<br>(11.0 to 17.8)
2	68.1<br>(62.8 to 72.9)	31.9<br>(27.1 to 37.2)	95.2<br>(93.2 to 96.7)	4.7<br>(3.3 to 6.8)	83.1<br>(78.7 to 86.8)	16.8<br>(13.2 to 21.3)
>2	73.2<br>(68.1 to 77.8)	26.8<br>(22.1 to 31.9)	96.3<br>(93.7 to 97.8)	3.7<br>(2.1 to 6.2)	86.0<br>(81.7 to 89.4)	14.0<br>(10.6 to 18.3)
Monthly plan premium (C+D)						
Zero dollar	68.5	31.5	96.3	3.7	87.5	12.5

© 2023 Gupta A et al. *JAMA Health Forum*.

	DENTAL<br>N= 7,516<br>Weighted % (95% CI)		VISION<br>N=8,026<br>Weighted % (95% CI)		HEARING<br>N=8,131<br>Weighted % (95% CI)	
**Enrolled in a MA plan→**	**with dental benefit**	**without dental benefit**	**with eye benefit**	**without eye benefit**	**with hearing benefit**	**without hearing benefit**
	(63.1 to 73.4)	(26.6 to 36.9)	(95.0 to 97.3)	(2.7 to 4.9)	(83.8 to 90.4)	(9.5 to 16.2)
≤$51.00	70.7 (64.0 to 76.7)	29.3 (23.3 to 36.0)	94.5 (89.4 to 97.1)	5.5 (2.8 to 10.6)	81.0 (72.9 to 86.8)	19.2 (13.1 to 27.1)
>51.00	64.7 (54.2 to 74.0)	35.3 (25.9 to 45.8)	95.1 (91.6 to 97.2)	4.9 (2.8 to 8.4)	81.7 (75.5 to 86.5)	18.3 (13.4 to 24.4)
Annual Plan OOP max[g]						
<$3,500	79.2 (73.4 to 84.0)	20.8 (15.9 to 26.6)	98.1 (95.3 to 99.3)	1.8 (0.7 to 4.8)	92.2 (85.7 to 95.9)	7.8 (4.1 to 14.3)
$3,501 - $4,950	67.4 (60.8 to 73.3)	32.6 (26.6 to 39.2)	96.4 (89.4 to 98.8)	3.6 (1.1 to 10.6)	83.8 (75.6 to 89.7)	16.1 (10.3 to 24.4)
$4,951 - $6,700	69.1 (59.3 to 77.5)	30.8 (22.4 to 40.7)	94.7 (92.0 to 96.6)	5.2 3.4 to 7.9)	84.9 (78.6 to 89.6)	15.1 (10.4 to 21.4)
>$6,700	60.8 (52.8 to 68.2)	39.2 (31.7 to 47.2)	94.6 (91.1 to 96.8)	5.3 (3.2 to 8.9)	81.5 (76.9 to 85.3)	18.5 (14.6 to 23.1)
Plan star rating						
<4	63.8 (56.5 to 70.6)	36.1 (29.4 to 43.5)	92.9 (89.9 to 95.2)	7.0 (4.8 to 10.1)	81.1 (75.9 to 85.4)	18.8 (14.6 to 24.0)
≥4	69.2 (64.3 to 73.8)	30.7 (26.2 to 35.8)	96.6 (94.8 to 97.8)	3.4 (2.2 to 5.2)	86.3 (81.7 to 89.8)	13.7 (10.2 to 18.2)
Type of coverage						
HMO	67.5 (61.7 to 72.7)	32.5 (27.3 to 38.2)	97.0 (94.0 to 98.6)	3.0 (1.4 to 5.9)	87.1 (82.6 to 90.5)	12.9 (9.4 to 17.3)
PPO/PFFS	71.1	28.9	92.6	7.4	81.3	18.7

© 2023 Gupta A et al. *JAMA Health Forum*.

	DENTAL<br>N= 7,516<br>Weighted % (95% CI)		VISION<br>N=8,026<br>Weighted % (95% CI)		HEARING<br>N=8,131<br>Weighted % (95% CI)	
**Enrolled in a MA plan→**	**with dental benefit**	**without dental benefit**	**with eye benefit**	**without eye benefit**	**with hearing benefit**	**without hearing benefit**
	(65.2 to 76.3)	(23.7 to 34.8)	(89.2 to 95.0)	(5.0 to 10.8)	(75.9 to 85.6)	(14.3 to 24.0)

Abbreviations: FPL: Federal Poverty Level; ADL: Activities of Daily Living; IADL: Instrumental Activities of Daily Living; OOP: Out-of-pocket; HMO: Health Maintenance Organization; PPO/PFFS: Preferred Provider Organization/Private Fee-for-Service. Weighted percentages represent the row percentage; [a]Not significantly different, all other associations were significantly different at P<0.001; [b]Education missing for 1.9%; [c] Marital status missing for 0.1%; [d]Health status missing for 4.7%; [e]Functional limitation missing for 4.5%; [f]Chronic condition missing for 4.4%; [g]Plan out-of-pocket maximum missing for 1.4%; row percentages may not add to 100% because of rounding; Other/multiple race/ethnicity category included those identifying as Asian or North American Natives or multiple race/ethnicities.

© 2023 Gupta A et al. *JAMA Health Forum*.

eTable 4. Adjusted Percentage-Point Difference in Beneficiary Enrollment by Dental Benefits, 2018 to 2020 Medicare Current Beneficiary Survey

	**Any dental benefit**		**Comprehensive dental benefit**	
	**Percentage point difference (95% CI)**	**P value**	**Percentage point difference (95% CI)**	**P value**
Race/ethnicity				
White	Ref		Ref	
Black	9.0 (3.4 to 14.4)	0.001	11.2 (5.7 to 16.7)	<0.001
Hispanic	2.8 (-5.7 to 11.4)	0.51	4.6 (-3.0 to 12.3)	0.23
Other	-5.6 (-14.4 to 3.2)	0.20	-3.8 (-13.2 to 5.6)	0.42
Income level				
>200%FPL	Ref		Ref	
≤200% FPL	-0.2 (-3.4 to 3.1)	0.91	4.4 (0.1 to 7.9)	0.01
Education				
College or higher	Ref		Ref	
Less than college	3.7 (0.1 to 7.4)	0.05	4.7 (1.4 to 8.0)	0.005
Health status				
Good/excellent	Ref		Ref	
Fair/poor	-0.8 (-4.5 to 2.9)	0.67	3.2 (-1.1 to 7.5)	0.15
Number of chronic conditions				
0	Ref		Ref	
1	-0.8 (-4.1 to 2.5)	0.63	0.3 (-3.8 to 4.6)	0.87
2	-1.3 (-5.3 to 2.7)	0.52	0.3 (-5.2 to 5.9)	0.90
>2	2.3 (-1.5 to 6.1)	0.22	3.1 (-2.8 to 8.9)	0.30
Functional limitations				
None	Ref		Ref	
Only IADL	2.4 (-0.8 to 5.5)	0.14	1.4 (-2.2 to 5.1)	0.44
1-2 ADL	-1.6 (-5.2 to 2.0)	0.39	0.2 (-3.6 to 4.1)	0.90
3-6 ADL	1.0 (-4.2 to 6.3)	0.70	-2.1 (-8.6 to 4.4)	0.52

© 2023 Gupta A et al. *JAMA Health Forum*.

eTable 5. Adjusted Percentage-Point Difference in Beneficiary Enrollment by Vision Benefits, 2018 to 2020 Medicare Current Beneficiary Survey

	**Any eye benefit**		**Eyewear benefit**	
	**Percentage point difference (95% CI)**	**P value**	**Percentage point difference (95% CI)**	**P value**
Race/ethnicity				
White	Ref		Ref	
Black	3.0 (0.1 to 5.0)	0.004	6.0 (0.6 to 11.5)	0.03
Hispanic	4.1 (1.1 to 7.0)	0.006	10.1 (1.4 to 18.7)	0.02
Other	-0.4 (-6.2 to 5.4)	0.88	11.5 (4.0 to 19.0)	0.003
Income level				
>200%FPL	Ref		Ref	
≤200% FPL	-1.5 (-2.9 to 0.0)	0.05	1.2 (-2.5 to 5.0)	0.51
Education				
College or higher	Ref		Ref	
Less than college	-1.4 (-3.0 to 0.2)	0.08	0.5 (-3.1 to 4.1)	0.77
Health status				
Good/excellent	Ref		Ref	
Fair/poor	0.2 (-1.6 to 2.1)	0.81	4.3 (-0.1 to 8.7)	0.05
Number of chronic conditions				
0	Ref		Ref	
1	-0.7 (-2.5 to 0.9)	0.39	-0.0 (-3.4 to 3.4)	0.99
2	-0.5 (-2.5 to 1.5)	0.64	-0.9 (-5.1 to 3.4)	0.68
>2	0.9 (-1.2 to 3.2)	0.40	3.5 (-1.5 to 8.5)	0.17
Functional limitations				
None	Ref		Ref	
Only IADL	-1.0 (-3.1 to 1.1)	0.35	-1.3 (-5.5 to 2.9)	0.54
1-2 ADL	-0.4 (-2.2 to 1.5)	0.69	-2.3 (-5.8 to 1.3)	0.20
3-6 ADL	-0.6 (-3.2 to 1.9)	0.61	-3.7 (-8.5 to 1.2)	0.14

© 2023 Gupta A et al. *JAMA Health Forum*.

eTable 6. Adjusted Percentage-Point Difference in Beneficiary Enrollment by Hearing Benefits, 2018 to 2020 Medicare Current Beneficiary Survey

	**Any hearing benefit**		**Hearing aid benefit**	
	**Percentage point difference (95% CI)**	**P value**	**Percentage point difference (95% CI)**	**P value**
Race/ethnicity				
White	Ref		Ref	
Black	2.8 (-0.9 to 6.6)	0.13	3.2 (-1.3 to 7.7)	0.17
Hispanic	0.7 (-6.2 to 7.6)	0.84	7.7 (-.7 to 14.8)	0.03
Other	-5.2 (-11.1 to 0.8)	0.09	-7.9 (-15.2 to -0.7)	0.03
Income level				
>200%FPL	Ref		Ref	
≤200% FPL	-0.2 (-2.4 to 1.9)	0.84	0.9 (-1.8 to 3.7)	0.49
Education				
College or higher	Ref		Ref	
Less than college	-1.5 (-4.2 to 1.2)	0.27	-0.1 (-3.1 to 3.0)	0.97
Health status				
Good/excellent	Ref		Ref	
Fair/poor	2.2 (-0.6 to 4.9)	0.12	0.2 (-3.8 to 4.2)	0.93
Number of chronic conditions				
0	Ref		Ref	
1	0.1 (-2.3 to 2.6)	0.92	1.5 (-1.4 to 4.5)	0.30
2	-2.2 (-5.6 to 1.0)	0.18	-0.6 (-4.6 to 3.3)	0.74
>2	1.3 (-1.5 to 4.3)	0.35	2.7 (-1.3 to 6.7)	0.19
Functional limitations				
None	Ref		Ref	
Only IADL	0.5 (-2.5 to 3.4)	0.76	3.0 (-0.7 to 6.7)	0.11
1-2 ADL	0.5 (-1.9 to 3.0)	0.67	2.5 (-0.5 to 5.5)	0.10
3-6 ADL	-1.9 (-6.9 to 2.9)	0.43	1.6 (-4.4 to 7.7)	0.60

© 2023 Gupta A et al. *JAMA Health Forum*.

eTable 7. Association Between Beneficiary Characteristics and Number of Dental, Vision, and Hearing Benefits, 2018 to 2020 Medicare Current Beneficiary Survey

	**Number of dental benefits**		**Number of vision benefits**		**Number of hearing benefits**	
	**IRR (95% CI)**	**P value**	**IRR (95% CI)**	**P value**	**IRR (95% CI)**	
Race/ethnicity						
White	Ref		Ref		Ref	
Black	1.2 (1.1 to 1.4)***	<0.001	1.0 (0.9 to 1.1)	0.75	1.0 (0.9 to 1.1)	0.11
Hispanic	1.1 (0.9 to 1.3)	0.25	1.0 (0.9 to 1.1)	0.60	1.0 (0.9 to 1.1)	0.21
Other	0.9 (0.7 to 1.1)	0.77	1.1 (0.9 to 1.1)	0.05	0.90 (0.8 to 1.0)*	0.02
Income level						
>200%FPL	Ref		Ref		Ref	
≤200% FPL	1.1 (0.9 to 1.1)	0.39	1.0 (0.9 to 1.0)	0.94	1.01 (0.9 to 1.0)	0.49
Education						
College or higher	Ref		Ref		Ref	
Less than college	1.1 (0.9 to 1.2)*	0.02	0.98 (0.9 to 1.0)	0.30	0.99 (0.96 to 1.0)	0.85
Sex						
Men	Ref		Ref		Ref	
Women	1.01 (0.9 to 1.1)	0.99	1.0 (1.0 to 1.1)*	0.02	1.0 (0.9 to 1.1)	0.07
Age group						
65-74 years	Ref		Ref		Ref	
75-84 years	0.98 (0.93 to 1.04)	0.07	0.95 (0.91 to 0.99)*	0.01	1.0 (0.9 to 1.1)	0.31
≥85 years	0.97 (0.92 to 1.04)	0.06	0.9 (0.9 to 1.0)	0.77	1.0 (0.9 to 1.1)	0.11
Marital status						
Married	Ref		Ref		Ref	
Not married	0.99 (0.93 to 1.01)	0.91	1.02 (0.9 to 1.05)	0.06	0.98 (0.95 to 1.0)	0.32
Rural-Urban residence						
Urban	Ref		Ref		Ref	
Rural	1.2 (0.8 to 1.6)	0.04	1.1 (0.9 to 1.2)	0.17	1.0 (0.9 to 1.1)	0.97
Health status						
Good/excellent	Ref		Ref		Ref	
Fair/poor	1.02 (0.9 to 1.1)	0.15	1.02 (0.9 to 1.1)	0.37	1.0 (0.9 to 1.1)	0.43
Number of chronic conditions						
0	Ref		Ref		Ref	
1	0.98 (0.91 to 1.1)	0.69	0.98 (0.95 to 1.0)	0.32	1.01 (0.9 to 1.04)	0.73
2	0.99 (0.90 to 1.1)	0.99	0.97 (0.92 to 1.0)	0.36	0.98 (0.94 to 1.0)	0.58
>2	1.02 (0.9 to 1.2)	0.59	1.01 (0.9 to 1.07)	0.48	1.0 (0.9 to 1.1)	0.40
Functional limitations						

© 2023 Gupta A et al. *JAMA Health Forum*.

	Number of dental benefits		Number of vision benefits		Number of hearing benefits	
	**IRR (95% CI)**	**P value**	**IRR (95% CI)**	**P value**	**IRR (95% CI)**	
None	Ref		Ref		Ref	
Only IADL	1.0 (0.9 to 1.1)	0.44	0.98 (0.94 to 1.0)	0.46	1.01 (0.9 to 1.1)	0.42
1-2 ADL	0.99 (0.91 to 1.1)	0.75	0.97 (0.93 to 1.0)	0.12	1.02 (0.9 to1.05)	0.37
3-6 ADL	0.99 (0.88 to 1.1)	0.97	0.96 (0.91 to 1.0)	0.15	0.99 (0.93 to 1.05)	0.76
Annual plan OOP maximum						
<$3,500	Ref		Ref		Ref	
$3,501 to $4,950	0.7 (0.5 to 1.0)	0.001	1.06 (1.0 to 1.12)*	0.04	0.8 (0.7 to 0.9)**	0.006
$4,951 to $6,700	0.7 (0.5 to 0.8)**	<0.001	1.00 (0.9 to 1.1)	0.99	0.8 (0.7 to 0.9)***	<0.001
>$6,700	0.6 (0.5 to 0.7)***	<0.001	0.91 (0.8 to 0.98)*	0.02	0.8 (0.7 to 0.9)***	<0.001
Monthly plan premium Part C & D						
Zero dollar	Ref		Ref		Ref	
$0.01 to $51.00	0.9 (0.8 to 1.1)	0.15	0.90 (0.8 to 0.97)**	0.003	0.9 (0.8 to 1.0)	0.18
>$51.00	0.8 (0.6 to 1.0)	0.01	1.02 (0.9 to 1.1)	0.45	0.84 (0.80 to 0.9)***	<0.001
Plan star rating						
<4 stars	Ref		Ref		Ref	
4 or above	0.9 (0.8 to 1.1)	0.42	1.1 (1.0 to 1.2)*	0.03	1.0 (0.9 to 1.1)	0.82
Coverage type						
HMO	Ref		Ref		Ref	
PPO/PFFS	1.3 (1.1 to 1.6)**	<0.001	0.9 (0.8 to 1.0)	0.07	0.9 (0.8 to 1.0)	0.10

Abbreviations: FPL: Federal Poverty Level; ADL: Activities of Daily Living; IADL: Instrumental Activities of Daily Living; OOP: Out-of-pocket; HMO: Health Maintenance Organization; PPO/PFFS: Preferred Provider Organization/Private Fee-for-Service

Incident Rate Ratios (IRR) were estimated from mixed-effects multivariable negative binomial regression models; *P<0.05; **P<0.01, ***P<0.001;

© 2023 Gupta A et al. *JAMA Health Forum*.

eAppendix. Sensitivity Analysis

Including county indicator instead of a county-level random intercept

In addition to race/ethnicity, we found that beneficiaries without a college degree (vs. higher educational attainment), were more likely to enroll in a plan with any dental benefit (5.0 ppd; 95% CI: 0.2 to 9.6; P<0.05). While race/ethnicity continued to be associated with dental comprehensive benefit, income and education were no longer significant. While Hispanic race/ethnicity continued to be associated with enrollment in a plan with any eye benefit (3.1 ppd; 95% CI: 0.2 to 6.1; P <0.05), Black and White beneficiaries were no longer different for enrollment in plan with an eye benefit. Results were similar to the primary analysis for eyewear and hearing benefits. We additionally found that as compared to those with no chronic condition, those with more than 2 chronic conditions were more likely to enroll in a plan with an eyewear benefit (4.5 ppd; 95% CI: 0.5 to 8.6; P<0.05) and the number of hearing benefits in a plan were not different between other/multiple vs. White race/ethnicity.

Adjusting for county level MA penetration instead of a county-level random effect

In addition to race/ethnicity, we found that beneficiaries without a college degree (vs. higher educational attainment), were more likely to enroll in a plan with any dental benefit (5.5 ppd; 95% CI: 0.8 to 10.2; P<0.05). Results were similar to the primary analysis for dental comprehensive benefit. While Hispanic race/ethnicity continued to be associated with enrollment in a plan with any eye benefit (3.2 ppd; 95% CI: 0.3 to 6.2; P<0.05), Black and White beneficiaries were no longer different for enrollment in plan with an eye benefit. Results were similar to the primary analysis for eyewear and hearing benefits. We additionally found that, as compared to those with no diagnosed chronic condition, those with more than 2 chronic conditions were more likely to enroll in a plan any an eyewear benefit (5.0 ppd; 95% CI: 1.1 to 8.9; P<0.05) and the number of hearing benefits in a plan were not different between other/multiple vs. White race/ethnicity.

Including beneficiaries age <65 years or enrolled in a C-SNP or I-SNP

In addition to race/ethnicity, we found that beneficiaries without a college degree (vs. higher educational attainment), were more likely to enroll in a plan with any dental benefit (5.9 ppd; 95% CI: 2.0 to 10.0; P<0.01). Results for enrollment in a plan with a dental comprehensive benefit or hearing benefits were similar to the primary analysis. Racial/ethnic minority groups were not different from White beneficiaries in their likelihood of enrolling in a plan with any eye or an eyewear benefit.

Including beneficiaries who are enrolled in a standalone plan for each benefit

In addition to race/ethnicity, beneficiaries without a college degree (vs. higher educational attainment), were more likely to enroll in a plan with any dental benefit (6.7 ppd; 95% CI: 2.2 to 11.3; P<0.01), or in plans with a higher number of dental benefits (IRR:1.1; 95% CI: 1.03 to 1.2; P<0.01). While other results were similar to the primary analysis, we found that Black (vs. White) beneficiaries were not significantly different in their likelihood of enrolling in a plan with any eye benefit, those with fair/poor health status (vs. good/excellent), were more likely to enroll in a plan any an eyewear benefit (4.2 ppd; 95% CI: 0.2 to 8.2; P<0.05) and Hispanic (vs. White) beneficiaries were more likely to enroll in a plan with a hearing aid benefit (8.5 ppd; 95% CI: 1.5 to 15.6; P<0.05).

Conditional logit models

© 2023 Gupta A et al. *JAMA Health Forum*.

Conditional logit models confirm our primary findings for dental benefits. We found that as compared to White beneficiaries, Black individuals, those with lower income and lower educational attainment had a higher odd of selecting a plan with a comprehensive dental benefit. Black individuals and those with lower educational attainment also had a higher odd of selecting a plan with any dental benefit.

For eye benefits, we found other/minority racial-ethnic groups to have higher odds of selecting a plan with an eyewear benefit. In the primary analysis we found Black and Hispanic respondents to also be more likely than White respondents to be enrolled in a plan with any eye or eyewear benefit. Also, while in the primary analysis, we found no difference in eye benefits by income and education, in conditional logit models we see that lower income and lower educated individuals are less likely to choose a plan with a vision benefit.

For hearing benefits, we find that Black (vs. White) respondents, and those with lower (vs. higher) income and lower (vs. higher) educational attainment had higher odds of selecting a plan with a hearing aid benefit. We did not find underserved groups to be more likely to choose a plan with a hearing benefit in our primary analysis, but conditional logit models show such a pattern of choosing plans with hearing benefits.

In the Table below, we are presenting the odds ratios from interaction terms between individual characteristics of interest (race/ethnicity, income, and education) and plan's dental, vision or hearing benefits, controlling for other plan characteristics.

	Black vs. White		Hispanic vs. White		Other/multiple vs. White		<=200% FPL vs. >200% FPL		<College degree vs. higher education	
Any dental benefit	1.5 (1.2 to 1.9)	0.001	0.9 (0.6 to 1.5)	0.967	0.5 (0.4 to 0.8)	0.002	1.1 (0.9 to 1.3)	0.071	1.3 (1.1 to 1.5)	0.001
Comprehensive dental benefit	1.6 (1.2 to 1.9	<0.001	1.1 (0.8 to 1.7)	0.532	0.5 (0.3 to 0.7)	<0.001	1.3 (1.2 to 1.5)	<0.001	1.3 (1.1 to 1.5)	<0.001
Any eye benefit	0.7 (0.5 to 1.5)	0.187	4.9 (0.7 to 35.7)	0.119	2.7 (0.8 to 8.9)	0.091	0.6 (0.5 to 0.8)	<0.001	0.5 (0.3 to 0.7)	<0.001
Eyewear benefit	0.9 (0.8 to 1.3)	0.979	1.8 (0.9 to 3.5)	0.057	2.4 (1.4 to 4.0)	0.001	1.1 (0.9 to 1.3)	0.342	0.8 (0.7 to 0.9)	0.036
Any hearing benefit	0.9 (0.7 to 1.3)	0.859	1.5 (0.8 to 2.9)	0.180	0.6 (0.4 to 0.9)	0.015	1.0 (0.9 to 1.2)	0.522	1.1 (0.9 to 1.3)	0.247
Hearing aid benefit	1.3 (1.1 to 1.7)	0.033	1.4 (0.9 to 2.3)	0.130	0.7 (0.5 to 1.1)	0.124	1.2 (1.1 to 1.3)	0.031	1.2 (1.0 to 1.4)	0.007

© 2023 Gupta A et al. *JAMA Health Forum*.
