# Supplementary material for: Enrollment Patterns of Medicare Advantage Beneficiaries by Dental, Vision, and Hearing Benefits
Source: JAMA Health Forum. 2024 Jan 12;5(1):e234936. doi: 10.1001/jamahealthforum.2023.4936 (PMC10787318; doi:10.1001/jamahealthforum.2023.4936)
Supplement: Supplement 2. — Data Sharing Statement [file jamahealthforum-e234936-s002.pdf]

Data Sharing Statement

Gupta. Enrollment Patterns of Medicare Advantage Beneficiaries by Dental, Vision, and Hearing Benefits. *JAMA Health Forum*. Published January 12, 2024. doi:10.1001/jamahealthforum.2023.4936

Data
Data available: Yes
Data types: Data (not involving human participants)
How to access data: This study uses the Limited Data Set (LDS) files from the Medicare Current Beneficiary Survey (MCBS), from years 2018 to 2020. The data can requested from the Centers for Medicare and Medicaid Services (CMS) at https://www.cms.gov/research-statistics-data-and-systems/files-for-order/limiteddatasets. The study also uses the Medicare Advantage benefit and enrollment files which are publicly available at https://www.cms.gov/research-statistics-data-and-systems/statistics-trends-and-reports/mcradvpartdenroldata.
When available: With publication

Supporting Documents
Document types: None

Additional Information
Who can access the data: The CMS requires a Data User Agreement to use MCBS LDS files and data can only be made available to researchers whose proposed use of the data has been approved by CMS.
Types of analyses: Analysis approved by CMS.
Mechanisms of data availability: After approval of a proposal and data user agreement with CMS.
